# Supplementary material for: Subclinical hypothyroidism in Wales from 2000 to 2021: A descriptive cohort study based on electronic health records
Source: PLoS One. 2024 May 21;19(5):e0298871. doi: 10.1371/journal.pone.0298871 (PMC11108130; doi:10.1371/journal.pone.0298871)
Supplement: S1 Appendix — (DOCX) [file pone.0298871.s001.docx]

**S1 Appendix. Description of SAIL datasets**

Demographic data, including residential periods in Wales, as well as indices of deprivation, are available from WDSD. All relevant GP records within the study timespan are obtained from WLGP – individual visits, investigations, diagnoses and prescribed medications per patient. Similarly, PEDW contains data on secondary care from all hospitals in Wales, precisely hospital admissions, diagnoses and interventions. Lab test result data across Wales are available from WRRS, regardless of whether the originator of the pathology request works in primary or secondary care. Outpatient referrals from primary care and the details of outpatient encounters are recorded in OPRD and OPDW, respectively. In EDDS, data are routinely collected on interactions with hospital emergency departments – this dataset was included to lessen the likelihood that patient outcomes would be missed. Deaths are recorded in multiple datasets, including WDSD, which contains demographic records and ADDE, a standalone register of all deaths in Wales. All EHR collected between 1 January 2000 and 31 December 2021 were subsequently provisioned.

**S1 Table 1. Datasets provided by SAIL Databank for this study**

| **SAIL Datasets** | **Description** |
| --- | --- |
| Annual District Death Extract (ADDE) | Death records |
| Emergency Department Dataset (EDDS) | Accident & Emergency records |
| Outpatient Database for Wales (OPDW) | Outpatient attendance records |
| Outpatient Referrals Dataset (OPRD) | Outpatient referral records |
| Patient Episode Database for Wales (PEDW) | Hospital admission records |
| Welsh Demographic Service Dataset (WDSD) | Demographic data |
| Welsh Longitudinal General Practice (WLGP) | Primary care/GP records |
| Welsh Results Reports Service (WRRS) | Laboratory test records |
