# Supplementary material for: Subclinical hypothyroidism in Wales from 2000 to 2021: A descriptive cohort study based on electronic health records
Source: PLoS One. 2024 May 21;19(5):e0298871. doi: 10.1371/journal.pone.0298871 (PMC11108130; doi:10.1371/journal.pone.0298871)
Supplement: S2 Appendix — (DOCX) [file pone.0298871.s002.docx]

**S2 Appendix. Case definition (SCH patients)**

Eligible patients were identified using a combination of Read v2 and International Classification of Diseases version 10 (ICD-10) diagnostic codes in the WLGP and PEDW/OPDW datasets, respectively (S2 Table). Office for Population Censuses Surveys version 4 (OPCS-4) codes were also used in PEDW to identify interventions such as investigations and surgeries that patients had received.

**S2 Table 1. Diagnostic codes used to identify patients with subclinical hypothyroidism**

| **Code** | **Type** | **Description** |
| --- | --- | --- |
| C047.00 | Read v2 | Subclinical hypothyroidism |
| C0A5.00 | Read v2 | Subclinical iodine-deficiency hypothyroidism |
| E02X | ICD-10 | Subclinical iodine-deficiency hypothyroidism |
| E038 | ICD-10 | Borderline hypothyroidism |

Because SCH is diagnosed due to thyroid function tests, WRRS was also checked for patients meeting the criteria of high TSH and normal FT4 from blood specimens collected on the same day. The respective lab reference ranges were used, as recorded alongside the test results. The assumption was that these patients had tests indicative of SCH, regardless of whether their corresponding GP or hospital records contained the appropriate diagnostic codes. It was not possible, however, to include levothyroxine treatment as a criterion for case identification because its use is not restricted to SCH.
