# Supplementary material for: Subclinical hypothyroidism in Wales from 2000 to 2021: A descriptive cohort study based on electronic health records
Source: PLoS One. 2024 May 21;19(5):e0298871. doi: 10.1371/journal.pone.0298871 (PMC11108130; doi:10.1371/journal.pone.0298871)
Supplement: S3 Appendix — (DOCX) [file pone.0298871.s003.docx]

**S3 Appendix. Age- and sex-stratified annual cumulative incidence according to the mid-2011 Welsh census data**


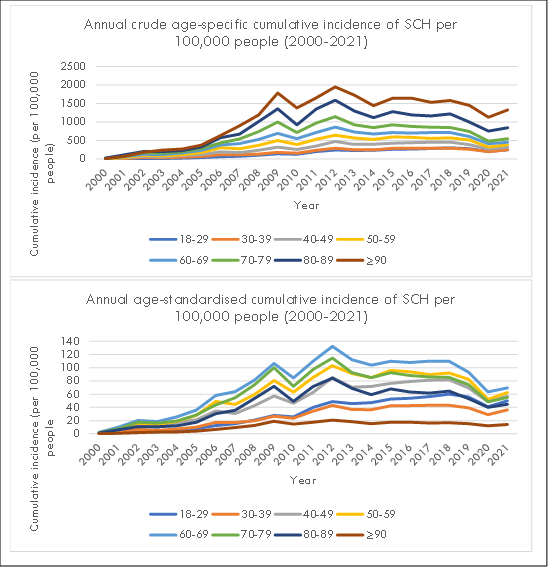


**S3 Fig 1. Age-standardised cumulative incidence of SCH over the study period (2000-2021)**


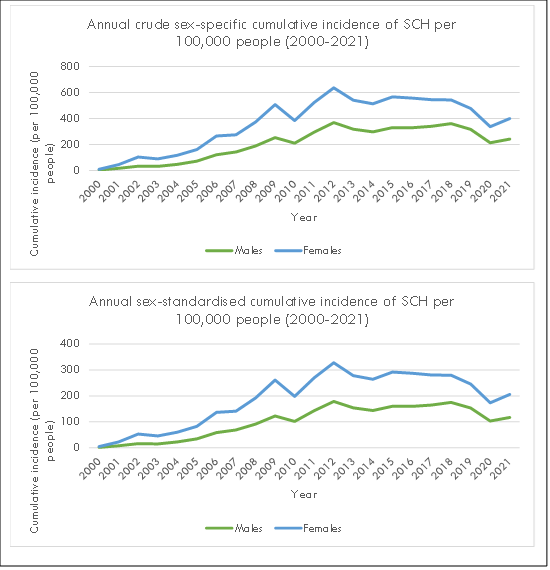


Supplementary Fig 2. Sex- standardised cumulative incidence of SCH over the study period (2000-2021)
