# Supplementary material for: Subclinical hypothyroidism in Wales from 2000 to 2021: A descriptive cohort study based on electronic health records
Source: PLoS One. 2024 May 21;19(5):e0298871. doi: 10.1371/journal.pone.0298871 (PMC11108130; doi:10.1371/journal.pone.0298871)
Supplement: S4 Appendix — (DOCX) [file pone.0298871.s004.docx]

**S4 Appendix. Levothyroxine use during the study period**

**S4 Table 1. Frequency counts of patients receiving levothyroxine over the study period (2000-2021)**

| Year | Cumulative total of SCH patients | Number of patients receiving LT4 prescriptions | | |
| --- | --- | --- | --- | --- |
|  |  | **Total (%)^a^** | **New users** | **Existing users** |
| 2000 | 200 | 52 (26.0) | 52 | 0 |
| 2001 | 1,143 | 187 (16.4) | 140 | 47 |
| 2002 | 3,235 | 773 (23.9) | 590 | 183 |
| 2003 | 5,103 | 1,333 (26.1) | 599 | 734 |
| 2004 | 7,669 | 2,126 (27.7) | 858 | 1,268 |
| 2005 | 11,307 | 3,180 (28.1) | 1,150 | 2,030 |
| 2006 | 17,392 | 4,673 (26.9) | 1,640 | 3,033 |
| 2007 | 23,969 | 5,815 (24.3) | 1,393 | 4,422 |
| 2008 | 32,892 | 7,302 (22.2) | 1,780 | 5,522 |
| 2009 | 44,962 | 9,340 (20.8) | 2,342 | 6,998 |
| 2010 | 54,396 | 11,160 (20.5) | 2,272 | 8,888 |
| 2011 | 67,382 | 13,321 (19.8) | 2,674 | 10,647 |
| 2012 | 83,353 | 16,014 (19.2) | 3,274 | 12,740 |
| 2013 | 97,011 | 18,398 (19.0) | 3,199 | 15,199 |
| 2014 | 109,942 | 20,775 (18.9) | 3,253 | 17,522 |
| 2015 | 124,291 | 23,105 (18.6) | 3,348 | 19,757 |
| 2016 | 138,509 | 25,549 (18.4) | 3,535 | 22,014 |
| 2017 | 152,754 | 27,784 (18.2) | 3,479 | 24,305 |
| 2018 | 167,333 | 29,764 (17.8) | 3,374 | 26,390 |
| 2019 | 180,184 | 31,534 (17.5) | 3,276 | 28,258 |
| 2020 | 189,103 | 32,185 (17.0) | 2,239 | 29,946 |
| 2021 | 199,520 | 33,337 (16.7) | 2,637 | 30,700 |

^a^ Percentage of all SCH patients in the respective year; ^b^ Calculated as the number of prescriptions divided by the total number of patients that received them.

Abbreviations: *LT4* levothyroxine; *SCH* subclinical hypothyroidism.
