# Supplementary material for: Subclinical hypothyroidism in Wales from 2000 to 2021: A descriptive cohort study based on electronic health records
Source: PLoS One. 2024 May 21;19(5):e0298871. doi: 10.1371/journal.pone.0298871 (PMC11108130; doi:10.1371/journal.pone.0298871)
Supplement: Attachment — Submitted filename: Response to Reviewers.docx [file pone.0298871.s005.docx]

13 January 2024

Dear Dr Ata,

**RE: Manuscript revisions for ‘Subclinical hypothyroidism in Wales from 2000 to 2021: a descriptive cohort study based on electronic health records’**

We would like to thank the editor and reviewers for their time and constructive feedback on our manuscript. In response to these comments, the quality of the manuscript has been improved as detailed in the table below.

All the manuscript changes can be viewed in the file labelled ‘Revised Manuscript with Track Changes' and a separate unmarked version, ‘Manuscript’, also provided. Both documents follow the PLOS ONE style requirements.

| **Reviewers' comments** | | **Line numbers (Tracked Changes)** |
| --- | --- | --- |
| Reviewer #1: | The demographic and clinical characteristics of patients diagnosed with subclinical hypothyroidism in Wales between 2000 and 2021 are described in this study. Furthermore, the annual cumulative incidence during this period and the testing and treatment patterns linked to the disorders were investigated. Although the sample size of the study was relatively large and the results obtained in this study provided considerable significance for the assessment of subclinical hypothyroidism, the authors should address the following requirements.  1. Some of the tables need to be changed as bar graphs or pie charts.  **Response:**  *We thank Reviewer 1 for their feedback on the manuscript. Because there are already five figures included in our original submission, we have limited scope to create additional figures. However, we have converted Table 3, which showed the frequency of abnormal tests before levothyroxine was initiated, to a bar graph – this is now included as Figure 5.* | Lines 270 – 275 and Fig5.tiff |
|  | 2. There was too much redundancy in the conclusion section. The authors need to concisely summarize or itemize the results.  **Response:**  *The original length of the Conclusion section was 227 words – this has now been shortened to 210 words and rearranged to improve flow. It now reads:*  *“This descriptive study on SCH in Wales shows an uneven rise in the overall number of patients, TFT and levothyroxine use between 2000 and 2021, with the highest annual cumulative incidence in 2012 at 502 cases per 100,000 people.*  *Compared to patients who only had test results indicative of SCH, those with clinically recorded diagnoses were less likely to meet the 10mIU/L TSH cutoff for severe SCH but were also more likely to be offered treatment. In contrast, patients with mild SCH on their index test were less likely to receive levothyroxine. The clinical management of SCH was inconsistent with the recommendation to consider treatment only if a repeat test reveals TSH levels higher than 10mIU/L, considering that over a third of treated cases had only one prior abnormal test result. However, per the current NICE annual monitoring guidance, TFTs were ordered more frequently for treated than untreated patients.*  *Our findings show that more robust guidelines are needed to ensure the appropriate clinical management of patients with SCH. Given the potential for a continued rise in patient numbers and conflicting evidence on the impact of SCH on patients’ long-term health, more research is needed to inform strategies to improve the use of TFTs and levothyroxine for managing this thyroid disorder.”* | Lines 448 - 461 |
| Reviewer #2: | In this article Brenda S Bauer and collegues report an discreptive study about the characterisation and the medication of SCH patients which covers almost the whole population of Wales spanning two decades, this study is of some interest,which indicates that more robust guidelines and recommendations are needed to ensure the consistent and appropriate clinical management of patients identified as having SCH.  **Response:**  *We thank Reviewer 2 for their comments on the manuscript.* | - |

Yours sincerely,

Brenda Bauer

Amaya Azcoaga-Lorenzo

Utkarsh Agrawal

Adeniyi Francis Fagbamigbe

Colin McCowan
